# Supplementary material for: A Wnt-induced conformational phospho-switch in DVL3 controls association with Frizzled receptors and Wnt/β-catenin signaling
Source: Sci Adv. 2026 May 13;12(20):eaed8899. doi: 10.1126/sciadv.aed8899 (PMC13170675; doi:10.1126/sciadv.aed8899)
Supplement: Supplementary file 1 — Figs. S1 to S13 Legends for tables S1 to S3 [file sciadv.aed8899_sm.pdf]

Science Advances
AAAS

Supplementary Materials for

A Wnt-induced conformational phospho-switch in DVL3 controls association with Frizzled receptors and Wnt/β-catenin signaling

Miroslav Micka *et al.*

Corresponding author: Konstantinos Tripsianes, kostas.tripsianes@ceitec.muni.cz;
Vítězslav Bryja, bryja@sci.muni.cz

Sci. Adv. **12**, eaed8899 (2026)
DOI: 10.1126/sciadv.aed8899

The PDF file includes:

Figs. S1 to S13
Legends for tables S1 to S3

Other Supplementary Material for this manuscript includes the following:

Tables S1 to S3

Figure S1

Repetition 1

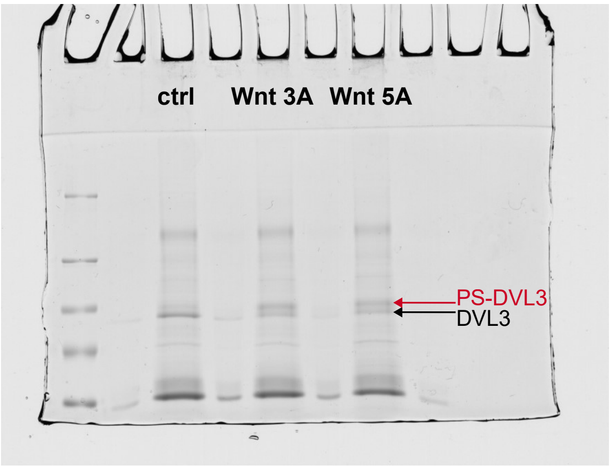

Repetition 2

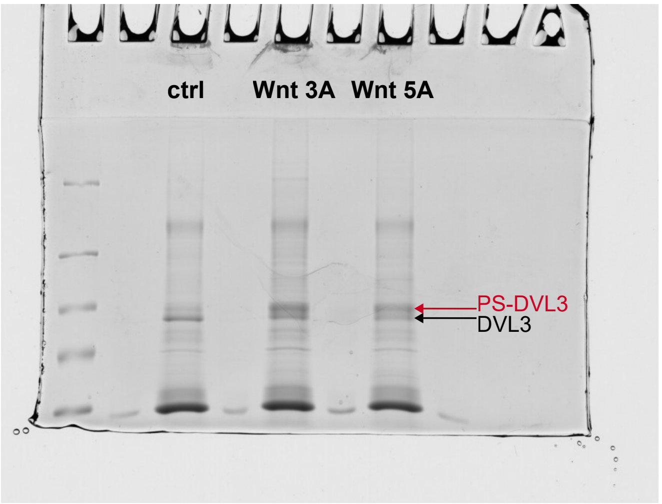

Repetition 3

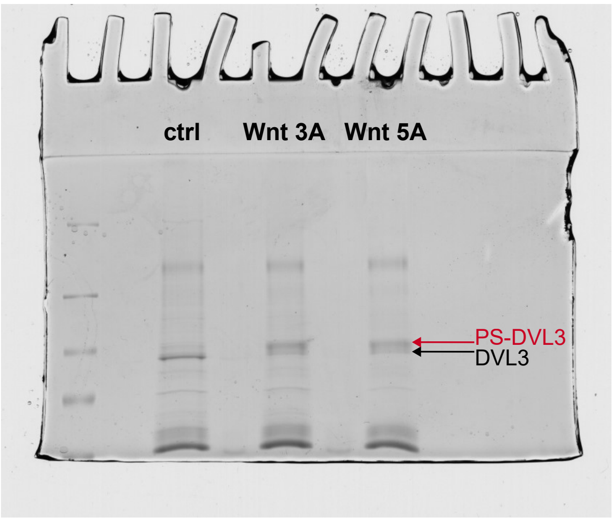

Figure S1. Gels used for the analysis of phosphorylation of the endogenous hDVL3. Black arrows represent hDVL3, while red arrows represent its phosphorylated state. The gel of Repetition 2 was used in **Fig 1A**.

Figure S2

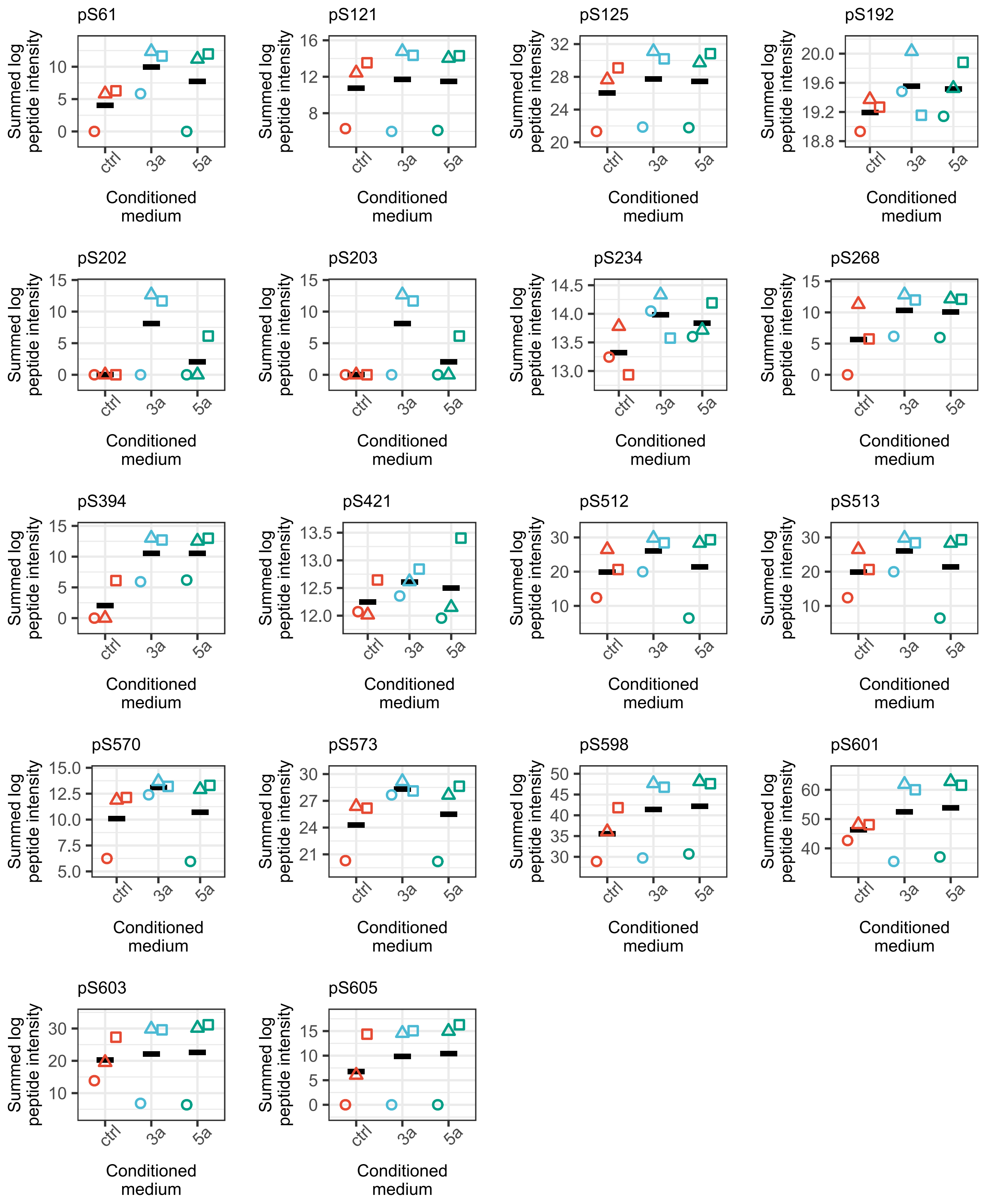

Figure S2. Plots of all detected phosphorylation sites in endogenous hDVL3. Different shapes represent three distinct biological replicates. The mean values are indicated by black dashes. The graph of pS394 is the same as in **Fig. 1C**.

Figure S3

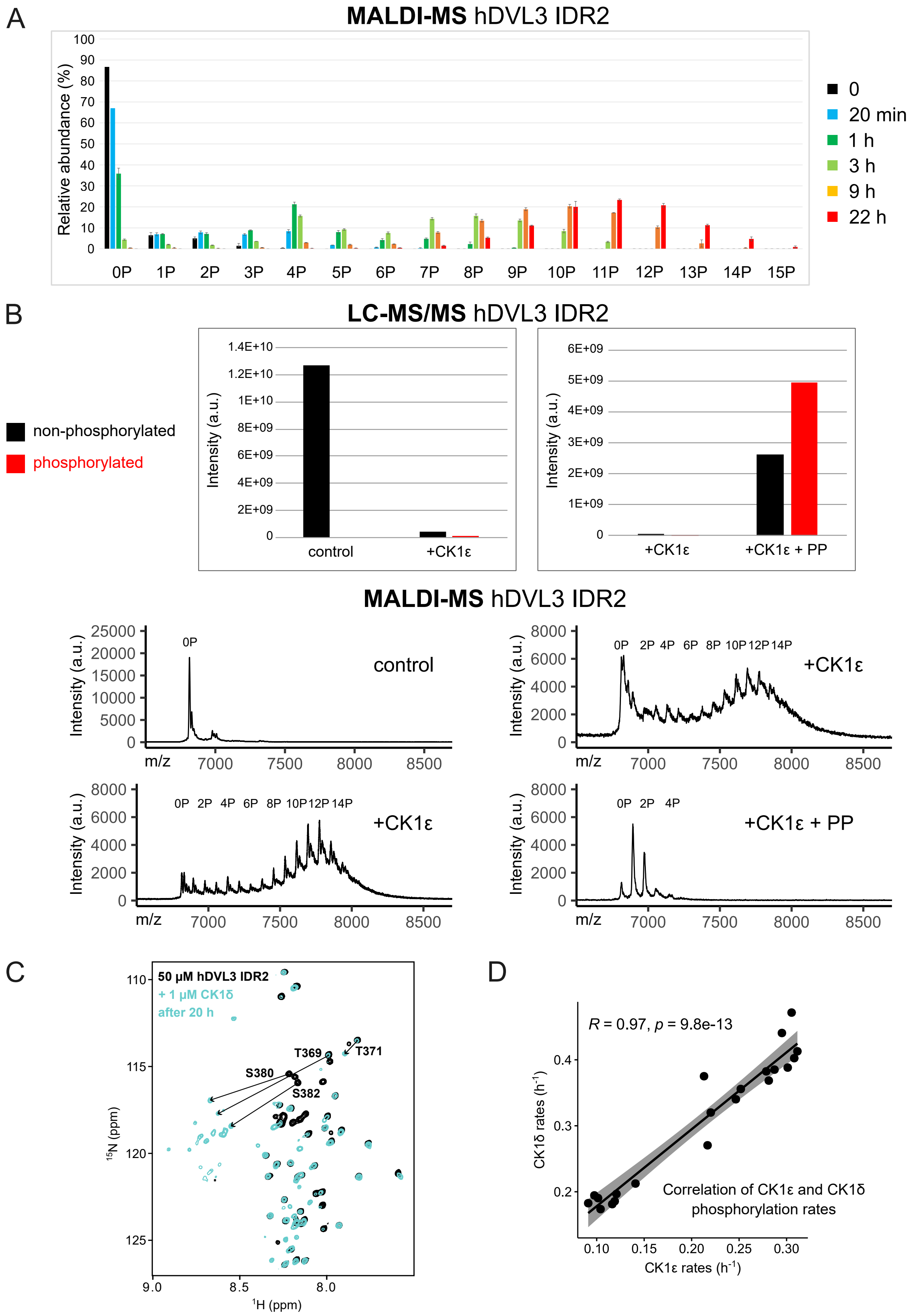
A
MALDI-MS hDVL3 IDR2
Relative abundance (%)
100
90
80
70
60
50
40
30
20
10
0
0P 1P 2P 3P 4P 5P 6P 7P 8P 9P 10P 11P 12P 13P 14P 15P
0
20 min
1 h
3 h
9 h
22 h
B
LC-MS/MS hDVL3 IDR2
non-phosphorylated
phosphorylated
Intensity (a.u.)
1.4E+10
1.2E+10
1E+10
8E+09
6E+09
4E+09
2E+09
0
control
+CK1ε
6E+09
5E+09
4E+09
3E+09
2E+09
1E+09
0
+CK1ε
+CK1ε + PP
MALDI-MS hDVL3 IDR2
25000
20000
15000
10000
5000
0
0P
control
m/z
7000
7500
8000
8500
8000
6000
4000
2000
0
0P 2P 4P 6P 8P 10P 12P 14P
+CK1ε
0P 2P 4P
+CK1ε + PP
C
50 µM hDVL3 IDR2
+ 1 µM CK1δ
after 20 h
T369
T371
S380
S382
110
115
120
125
^{15}N (ppm)
9.0
8.5
8.0
^{1}H (ppm)
D
R = 0.97, p = 9.8e-13
Correlation of CK1ε and CK1δ phosphorylation rates
CK1δ rates (h^{-1})
0.4
0.3
0.2
0.10
0.15
0.20
0.25
0.30
CK1ε rates (h^{-1})

Figure S3. In vitro multisite phosphorylation of hDVL3 IDR2 by CK1δ and CK1ε.
(**A**) Progressive accumulation of multisite-phosphorylated species of hDVL3 IDR2 over the CK1ε-mediated phosphorylation time course as determined by MALDI-MS. Each sample was analyzed four times, and the mean value was plotted. (**B**) LC-MS/MS detects the non-phosphorylated but not the phosphorylated peptides corresponding to the S/T cluster proximal to DEP. When the hDVL3 pIDR2 is dephosphorylated by protein phosphatase (PP) LC-MS/MS detects non-phosphorylated and phosphorylated peptides. MALDI-MS analysis of the same samples demonstrates that LC-MS/MS fails to detect highly phosphorylated peptides but is able to detect the corresponding peptides carrying 0, 1, or 2 phosphate groups after pIDR2 dephosphorylation. (**C**) Overlay of ^{1}H-^{15}N HSQC spectra of ^{15}N-labelled hDVL3 IDR2 before the reaction (black) and after 20 hours of in vitro phosphorylation by 1 μM CK1δ (cyan). Phosphorylation-induced changes in the chemical shift of S/T residues are highlighted by black arrows. (**D**) Correlation between CK1ε and CK1δ phosphorylation rates of individual IDR2 residues.

Figure S4

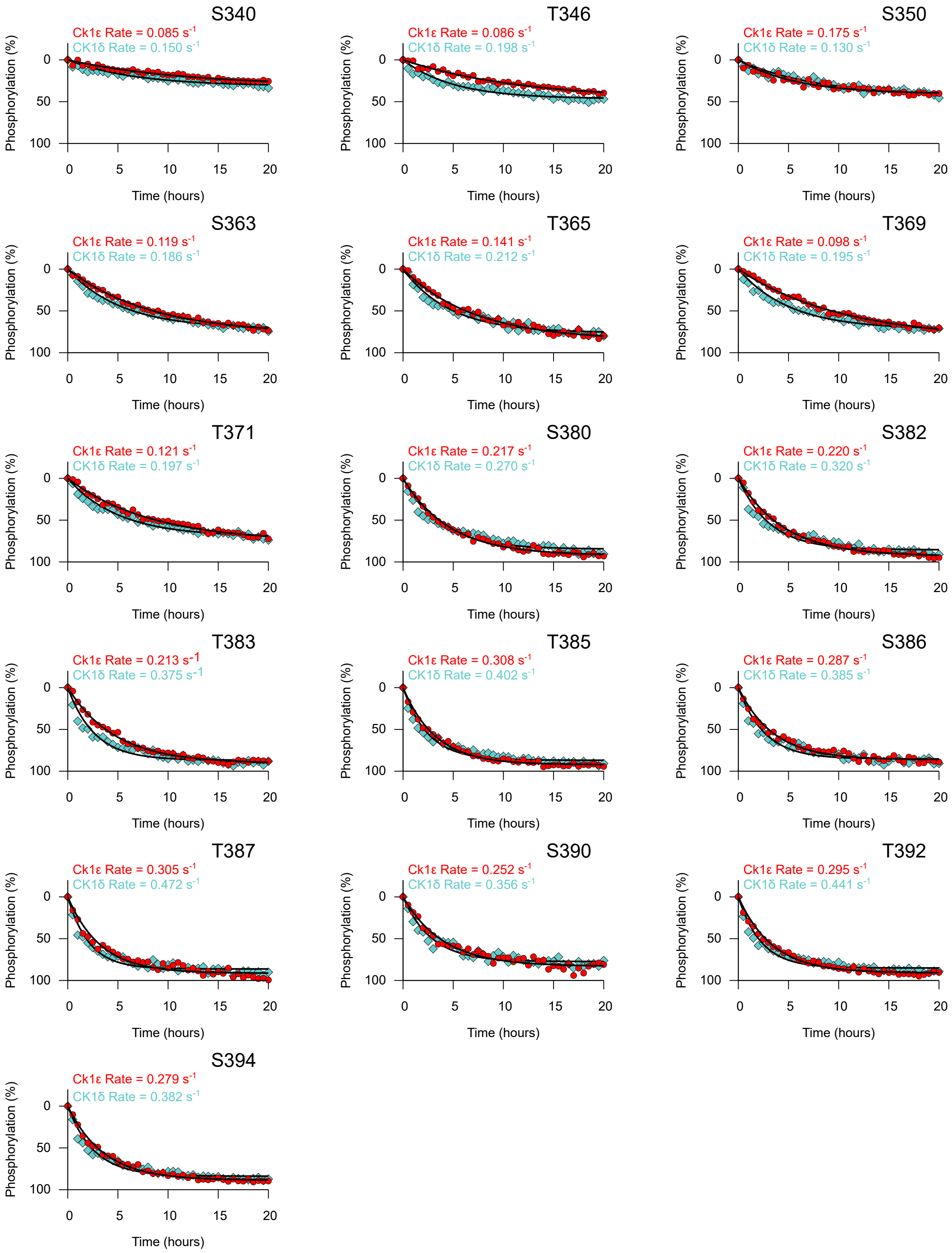

Figure S4. Graphs representing phosphorylation time course of individual S/T residues of hDVL3 IDR2.

Phosphorylated by CK1ε (red) or CK1δ (cyan). Kinetics were measured as the decay of the peak intensity of the non-phosphorylated S/T residues.

Figure S5

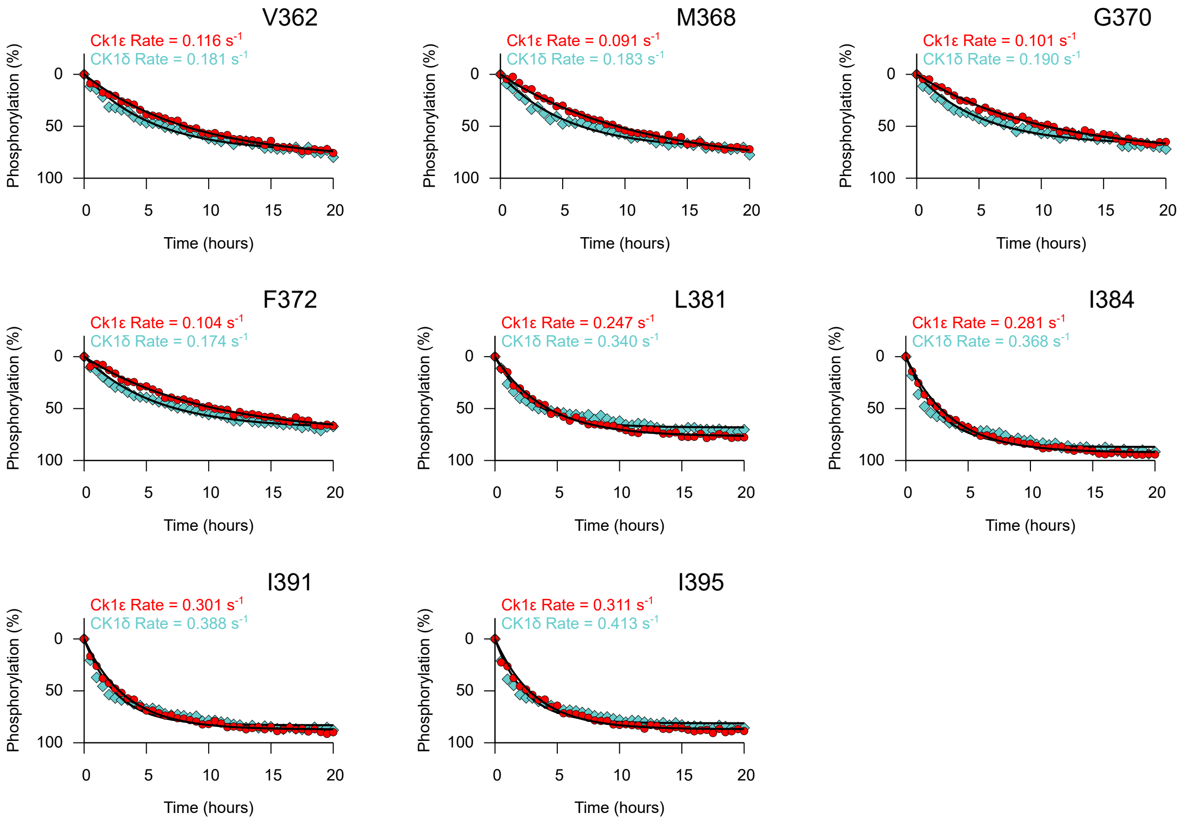

Figure S5. Graphs representing phosphorylation time course of residues neighboring S/T of hDVL3 IDR2.
Phosphorylated by CK1ε (red) or CK1δ (cyan). Kinetics were measured as the decay of the peak intensity of the non-phosphorylated residues.

Figure S6

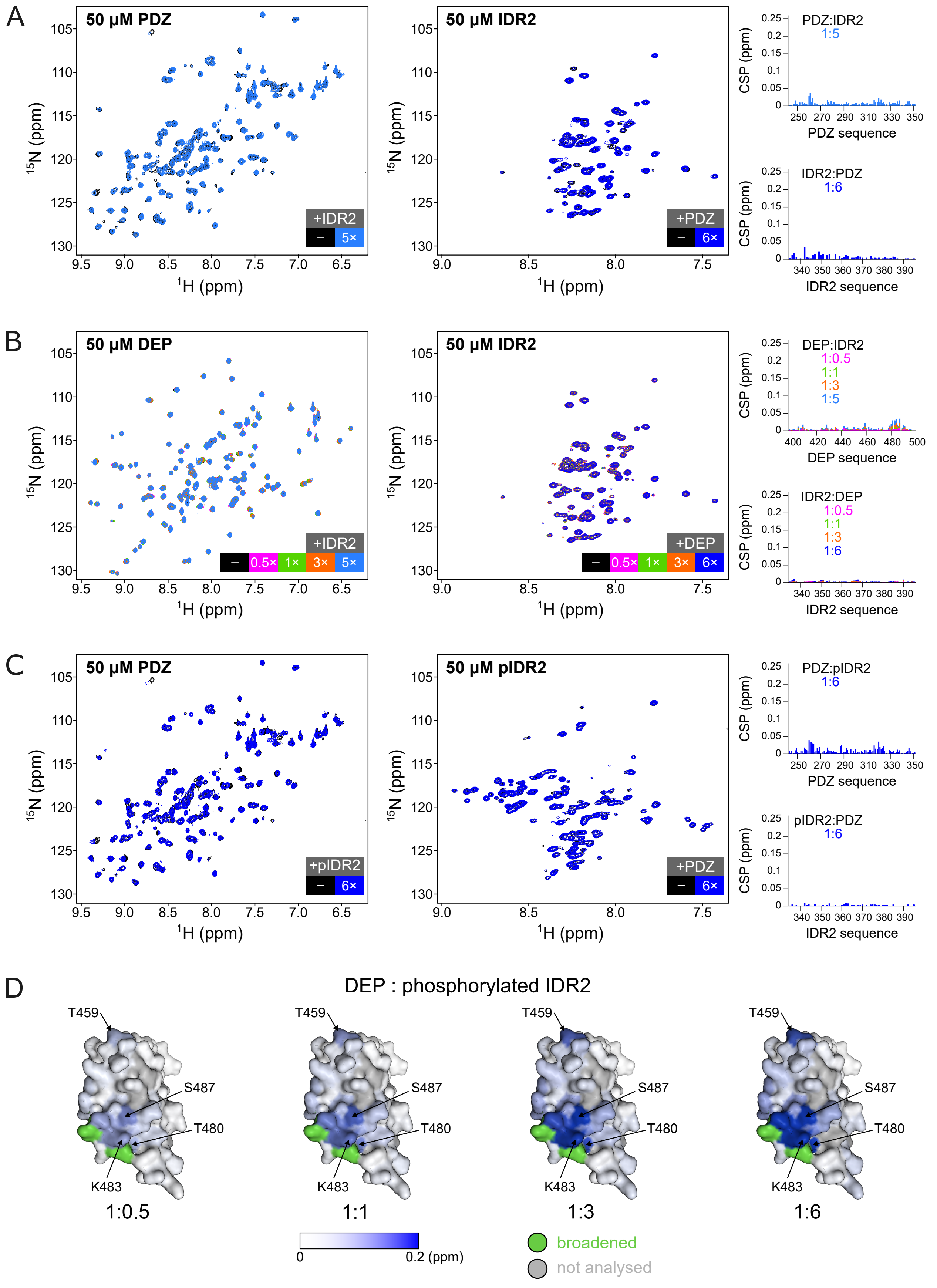

A
50 µM PDZ
+IDR2
–
5×
50 µM IDR2
+PDZ
6×
PDZ:IDR2
1:5
PDZ sequence
IDR2:PDZ
1:6
IDR2 sequence
B
50 µM DEP
+IDR2
0.5×
1×
3×
5×
50 µM IDR2
+DEP
DEP:IDR2
1:0.5
1:1
1:3
1:5
DEP sequence
IDR2:DEP
1:6
C
50 µM PDZ
+pIDR2
50 µM pIDR2
PDZ:pIDR2
pIDR2:PDZ
D
DEP : phosphorylated IDR2
T459
S487
T480
K483
1:0.5
1:1
1:3
1:6
0
0.2 (ppm)
broadened
not analysed

Figure S6. Analysis of IDR2 and pIDR2 interaction with the adjacent domains PDZ and DEP.

(**A**) Overlay of ^{1}H-^{15}N HSQC spectra of ^{15}N-labeled 50 μM hDVL3 PDZ domain (aa 243-351) free and in the presence of 250 μM hDVL3 IDR2 (aa 335-396). Overlay of ^{1}H-^{15}N HSQC spectra of ^{15}N-labeled 50 μM hDVL3 IDR2 free and in the presence of 300 μM hDVL3 PDZ. CSP quantification across the PDZ or IDR2 sequence, respectively. (**B**) Overlay of ^{1}H-^{15}N HSQC spectra of ^{15}N-labeled 50 μM hDVL3 DEP domain free and in the presence of increasing amounts of hDVL3 IDR2. Overlay of ^{1}H-^{15}N HSQC spectra of ^{15}N-labeled 50 μM hDVL3 IDR2 free and in the presence of increasing amounts of hDVL3 DEP. CSP quantification across the DEP or IDR2 sequence, respectively. (**C**) Overlay of ^{1}H-^{15}N HSQC spectra of ^{15}N-labeled 50 μM hDVL3 PDZ domain free and in the presence of 300 μM phosphorylated hDVL3 IDR2 (pIDR2). Overlay of ^{1}H-^{15}N HSQC spectra of ^{15}N-labeled 50 μM hDVL3 pIDR2 free and in the presence of 300 μM hDVL3 PDZ. CSP quantification across the PDZ or IDR2 sequence, respectively. (**D**) CSPs in the DEP spectrum, caused by the serial titration of pIDR2, mapped onto the DEP structure (see also **Fig. 4B**).

Figure S7

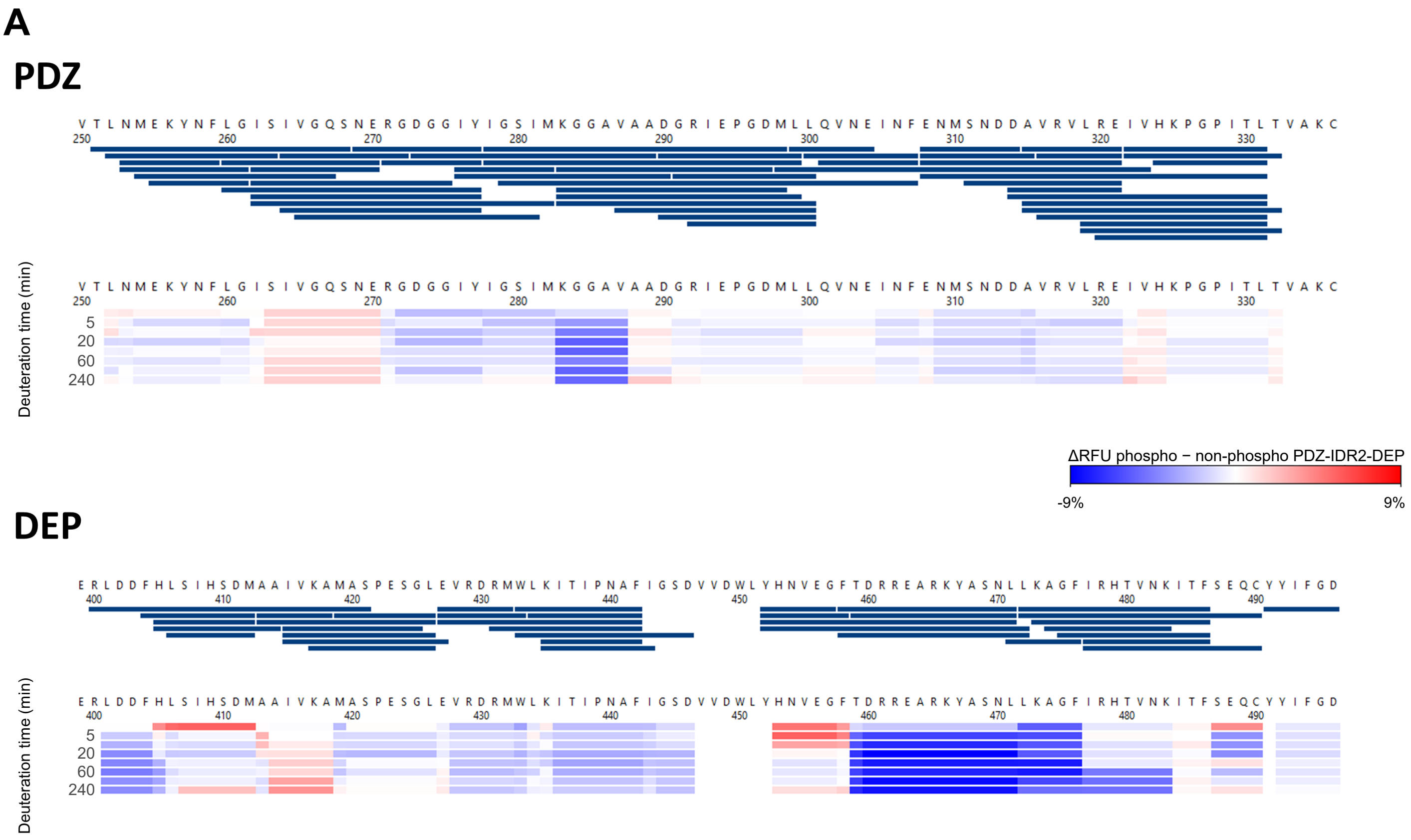

B

Data Set	phospho Dvl3	Dvl3_NoPhospho
HDX reaction details	10mM Tris, 100mM NaCl, pD 7.1	
Digestion Temperature	20°C	
HDX time course	0, 0.5, 5, 10, 20, 40, 60, 120, 240 mins	
# of Peptides	88	
Sequence coverage PDZ (240 - 338) :	82.83%	
Sequence coverage DEP (404 - 496) :	94.62%	
Average peptide length / Redundancy	12,16 / 6,29	
Replicates (technical)	3	3
Repeatability (average standard deviation)	0.0333	0.0366

Figure S7. Hydrogen-deuterium exchange (HDX) measurments.
(**A**) HDX-MS Coverage maps and heatmaps for the PDZ and DEP domains. Blue bars represent the identified peptides after data curation, and the heatmaps represent the percentual differential relative fractional uptake (phosphorylated – non-phosphorylated PDZ-IDR2-DEP) for all deuteration time points analyzed (top to bottom: 0.5, 5, 10, 20, 40, 60, 120, and 240 min). (**B**) Summary of HDX-MS measurments.

Figure S8

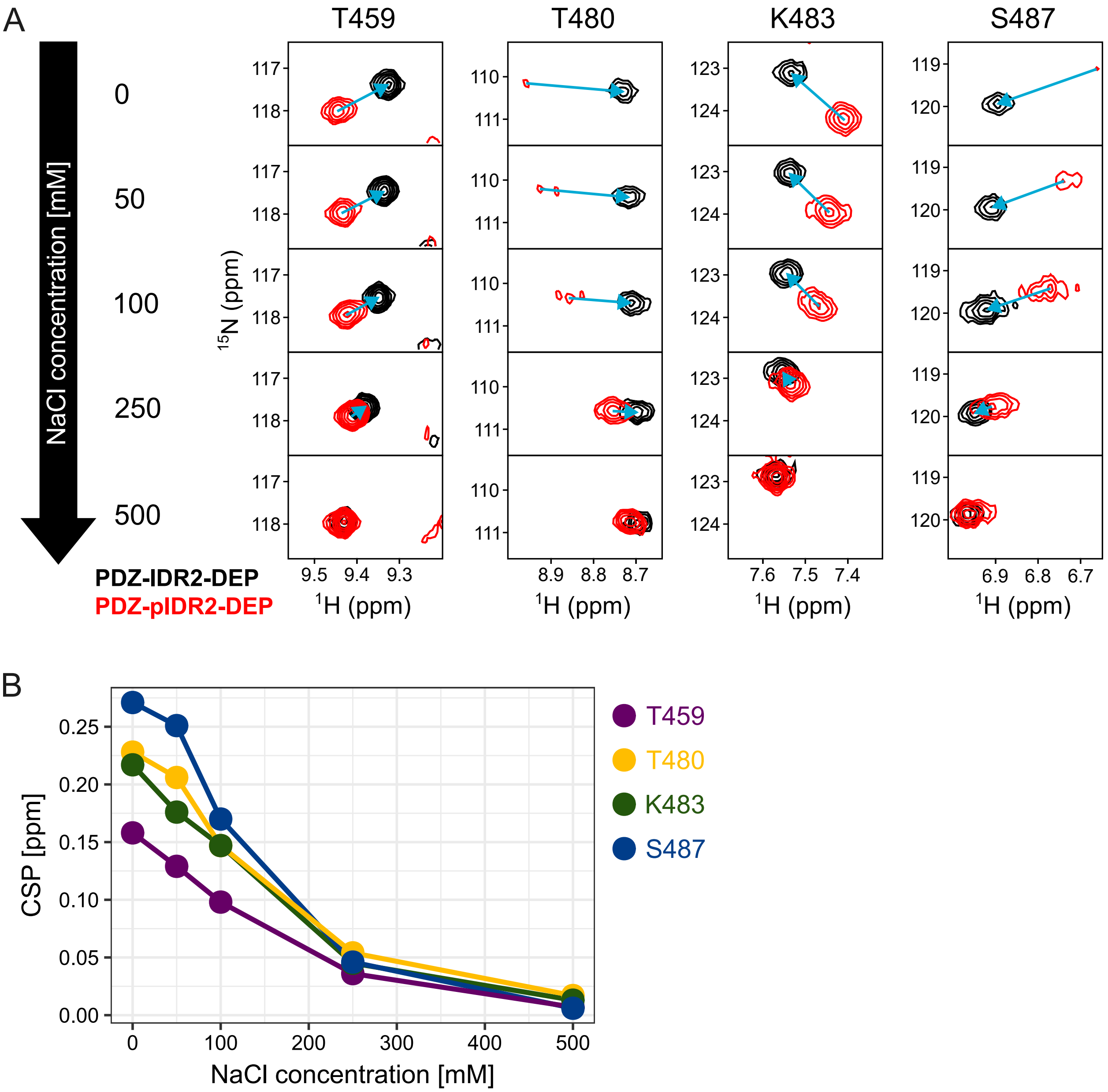

Figure S8. The intramolecular interaction between pIDR2 and DEP is electrostatic in nature.
(**A**) The DEP reporter peaks from the ^{1}H-^{15}N HSQC spectra of hDVL3 PDZ-IDR2-DEP (aa 243-496, black) overlaid with ^{1}H-^{15}N HSQC spectra of phosphorylated PDZ-IDR2-DEP (PDZ-pIDR2-DEP, red) in the presence of increasing NaCl concentrations. Arrows indicate the chemical shift perturbations (CSPs) of the selected residues caused by phosphorylation at different salt concentrations. (**B**) CSPs of the selected residues shown in **A** as a function of increasing NaCl concentration. The following concentrations were tested: 0 mM, 50 mM, 100 mM, 250 mM, and 500 mM NaCl.

Figure S9

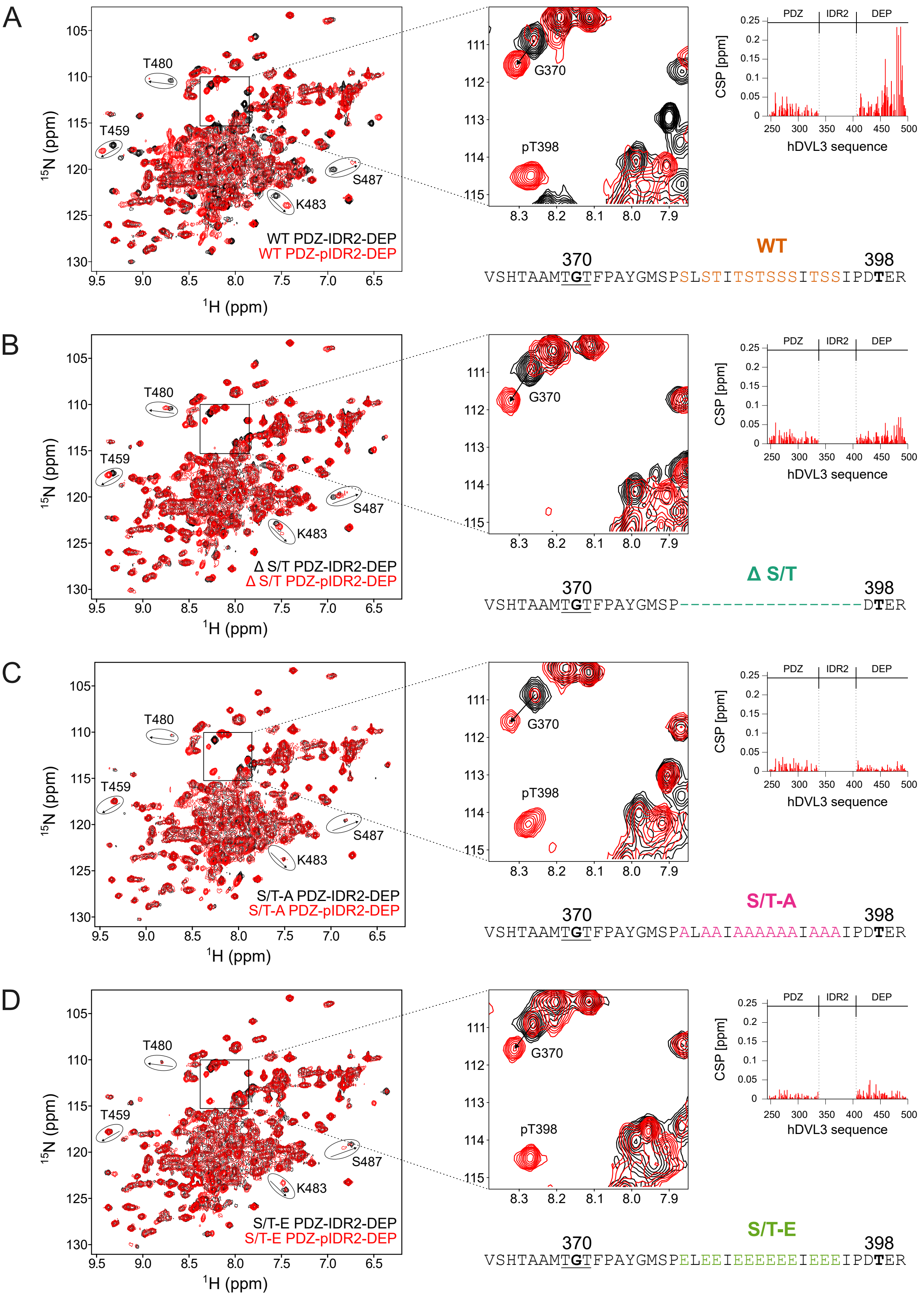
A
T480
T459
S487
K483
WT PDZ-IDR2-DEP
WT PDZ-pIDR2-DEP
^{15}N (ppm)
^{1}H (ppm)
G370
pT398
PDZ
IDR2
DEP
CSP [ppm]
hDVL3 sequence
WT
370
398
VSHTAAMTGTFPAYGMSPSLSTITSTSSSITSSIPDTER
B
T480
T459
S487
K483
Δ S/T PDZ-IDR2-DEP
Δ S/T PDZ-pIDR2-DEP
^{15}N (ppm)
^{1}H (ppm)
G370
PDZ
IDR2
DEP
CSP [ppm]
hDVL3 sequence
Δ S/T
370
398
VSHTAAMTGTFPAYGMSP----------------DTER
C
T480
T459
S487
K483
S/T-A PDZ-IDR2-DEP
S/T-A PDZ-pIDR2-DEP
^{15}N (ppm)
^{1}H (ppm)
G370
pT398
PDZ
IDR2
DEP
CSP [ppm]
hDVL3 sequence
S/T-A
370
398
VSHTAAMTGTFPAYGMSPALAAIAAAAAAIAAAIPDTER
D
T480
T459
S487
K483
S/T-E PDZ-IDR2-DEP
S/T-E PDZ-pIDR2-DEP
^{15}N (ppm)
^{1}H (ppm)
G370
pT398
PDZ
IDR2
DEP
CSP [ppm]
hDVL3 sequence
S/T-E
370
398
VSHTAAMTGTFPAYGMSPELEEIEEEEEEIEEEIPDTER

Figure S9. NMR analysis of WT and phospho-switch mutant series.
(**All panels**) Overlay of ^{1}H-^{15}N HSQC spectra of PDZ-IDR2-DEP and PDZ-pIDR2-DEP. DEP reporter peaks are circled and indicated by arrows. Zoom-in view highlights the G370 chemical shift change reporting on T369-T371 phosphorylation and shows annotated peak position of phosphorylated T398 (pT398). Phosphorylation-induced CSPs are plotted against the DVL3 sequence. (**A**) WT. (**B**) Δ S/T. (**C**) S/T-A. (**D**) S/T-E.

Figure S10

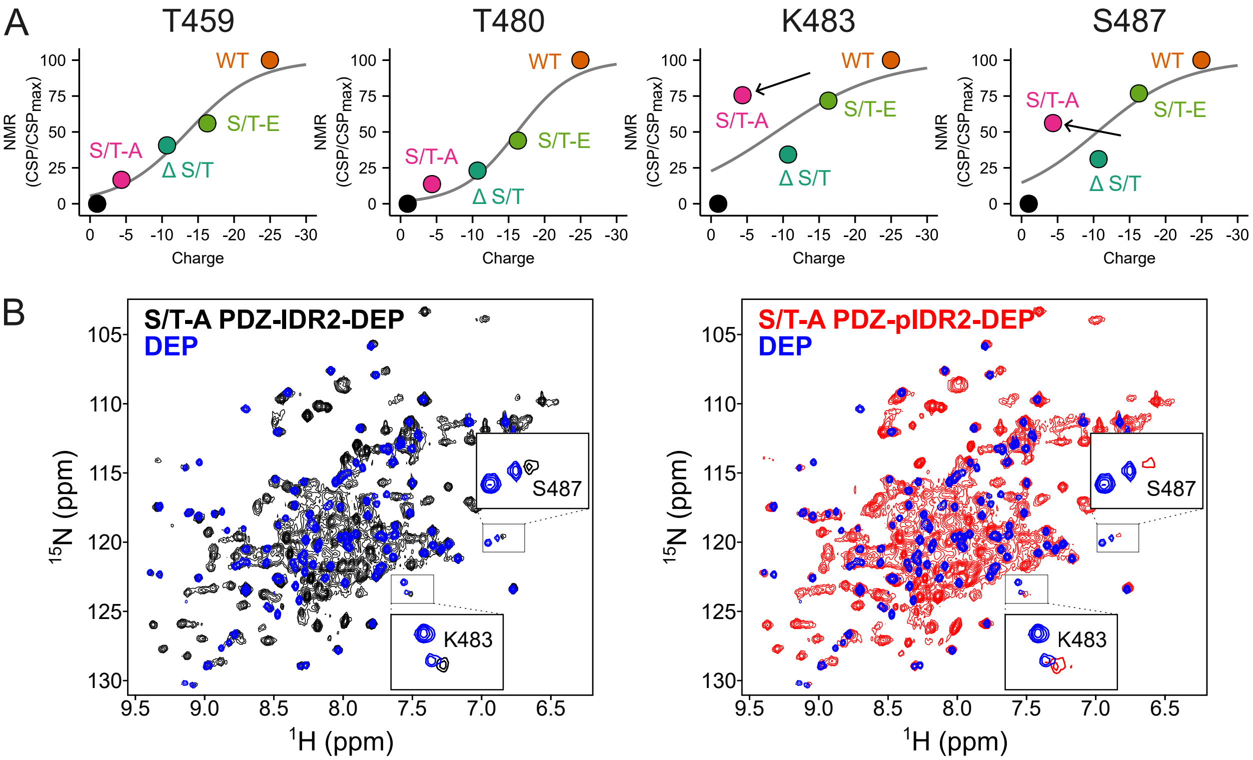

Figure S10. Intramolecular interaction measured by NMR as a function of net charge in the proximity of the DEP domain.
(**A**) Plots for individual reporter peaks T459, T480, K483, and S487, respectively, reporting on intramolecular interaction as a function of net charge proximal to DEP fitted to a sigmoidal function. (**B**) Overlay of ^{1}H-^{15}N HSQC spectra of S/T-A in non-phosphorylated and phosphorylated form and the DEP domain of WT DVL3. S/T-A peaks K483 and S487 report on the exchange phenomena observed in the DEP domain which are unrelated to IDR2 phosphorylation and the intramolecular interaction. Thus, the S/T-A data points for K483 and S487 indicated by arrows in **A** were not used in calculating the mean value of CSP/CSP_{max} in the corresponding plot in **Fig. 5C**.

Figure S11

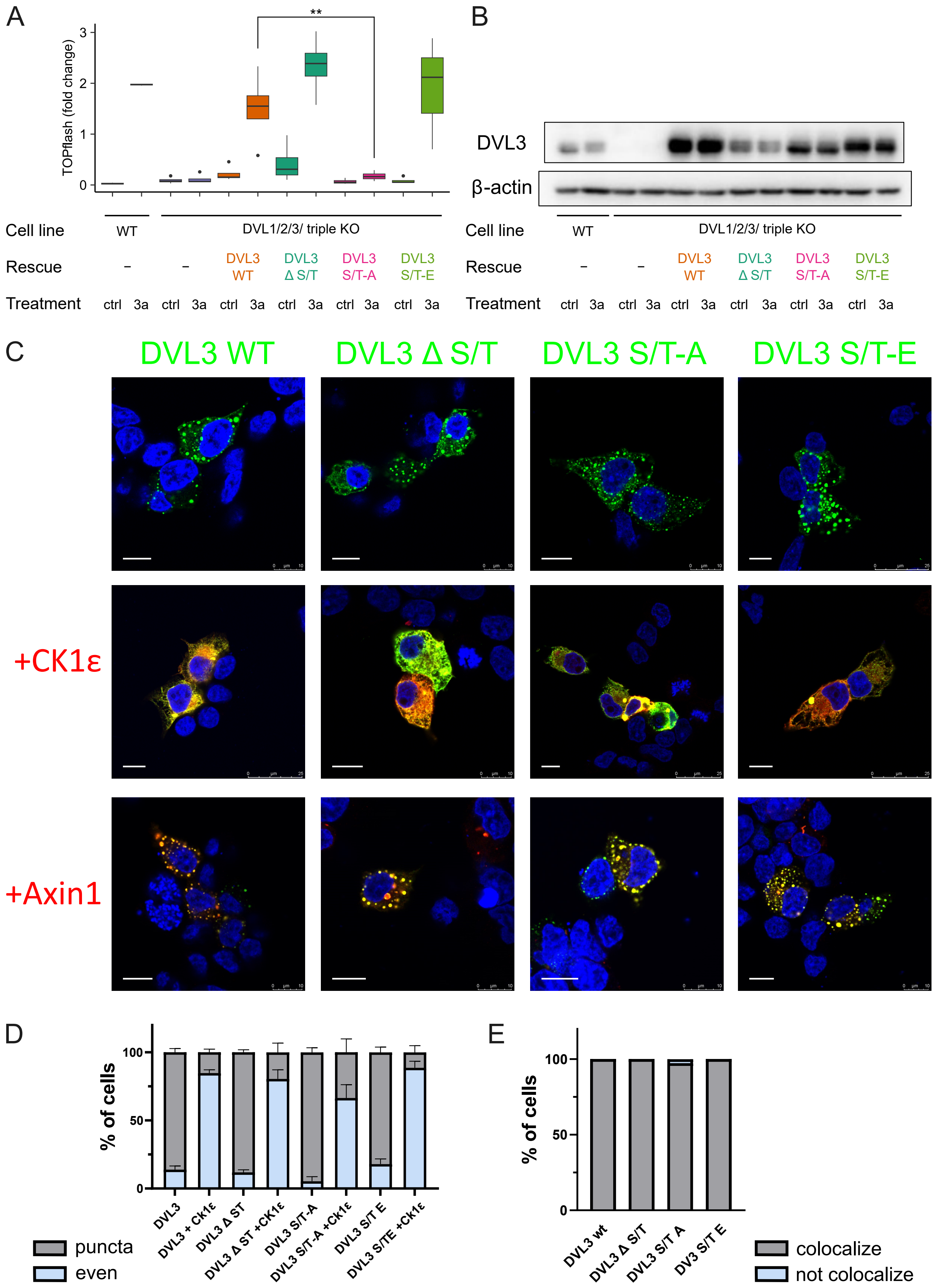
A
B
C
D
E
TOPflash (fold change)
**
DVL3
β-actin
Cell line
WT
DVL1/2/3/ triple KO
Rescue
DVL3 WT
DVL3 Δ S/T
DVL3 S/T-A
DVL3 S/T-E
Treatment
ctrl 3a ctrl 3a ctrl 3a ctrl 3a ctrl 3a ctrl 3a
DVL3 WT
DVL3 Δ S/T
DVL3 S/T-A
DVL3 S/T-E
+CK1ε
+Axin1
% of cells
DVL3
DVL3 + CK1ε
DVL3 Δ ST
DVL3 Δ ST +CK1ε
DVL3 S/T-A
DVL3 S/T-A +CK1ε
DVL3 S/T E
DVL3 S/TE +CK1ε
puncta
even
DVL3 wt
DVL3 Δ S/T
DVL3 S/T A
DV3 S/T E
colocalize
not colocalize

Figure S11. Cellular effects of the phospho-switch mutant series.

(**A**) TOPflash assay. Wild-type (WT) or DVL1/2/3 triple KO cells were treated with control (ctrl) or Wnt-3a (3a) conditioned media to induce signal transduction. Full length WT or phosphoswitch mutant series were reintroduced into the triple KO cells to rescue the signal. Statistical significance was tested by ANOVA. ** stands for 0.01 significance level. Four distinct biological replicates were analyzed. (**B**) Expression of hDVL3 variants used in **A**. β-actin was used as the loading control. (**C**) Representative pictures of the subcellular localization of hDVL3 mutant variants, overexpressed alone or together with CK1ε or Axin1, respectively. Scale bar = 10 μm (**D**). Quantification of the subcellular localization of hDVL3 mutant variants overexpressed alone or together with CK1ε, respectively. Three distinct biological replicates were analyzed. (**E**) Quantification of the colocalization of hDVL3 mutant variants with Axin1. N = 1.

Figure S12

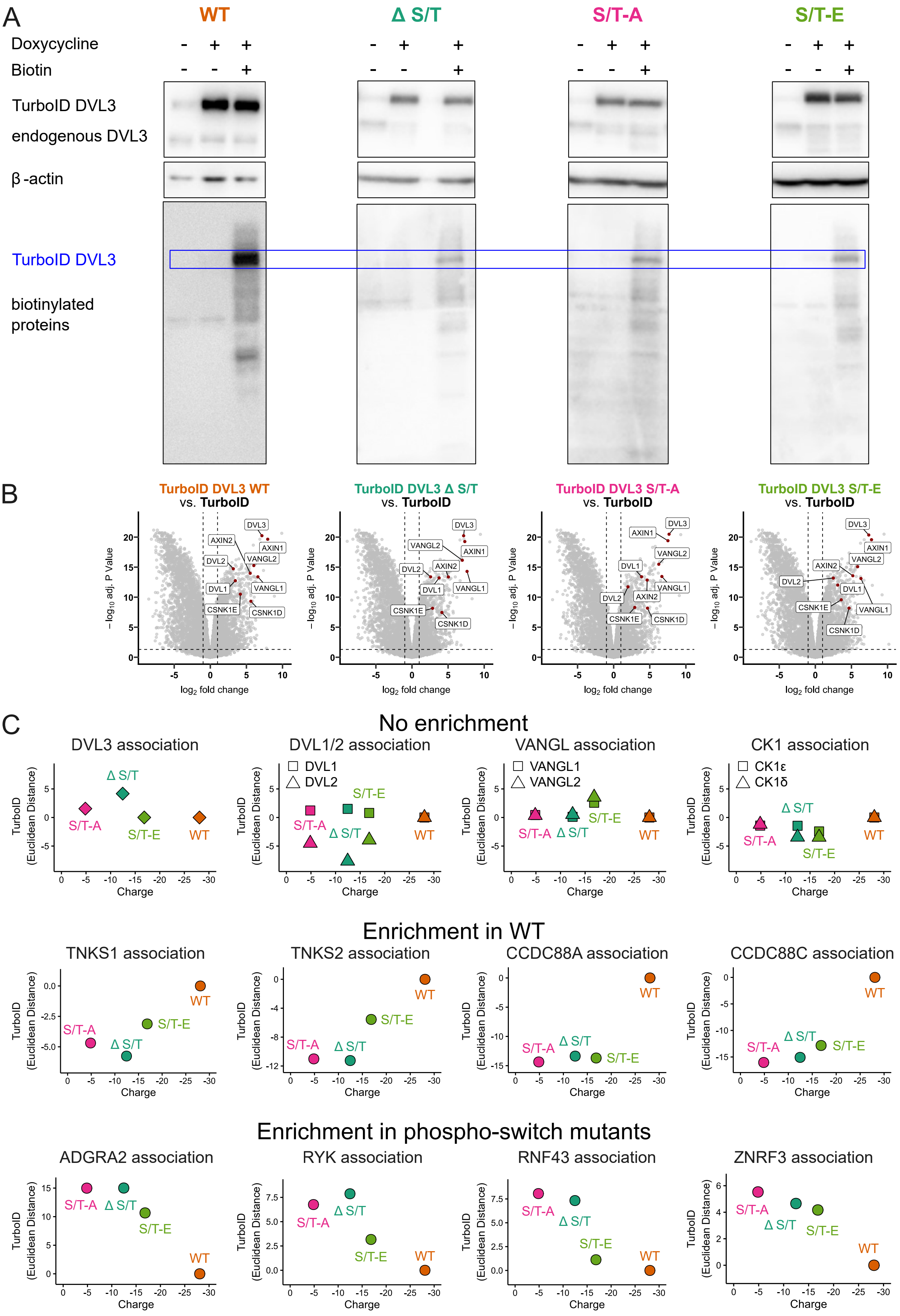

A
WT
Δ S/T
S/T-A
S/T-E
Doxycycline
Biotin
TurboID DVL3
endogenous DVL3
β-actin
TurboID DVL3
biotinylated proteins
B
TurboID DVL3 WT vs. TurboID
TurboID DVL3 Δ S/T vs. TurboID
TurboID DVL3 S/T-A vs. TurboID
TurboID DVL3 S/T-E vs. TurboID
DVL3
AXIN1
AXIN2
VANGL2
VANGL1
DVL1
DVL2
CSNK1E
CSNK1D
log2 fold change
−log10 adj. P Value
C
No enrichment
DVL3 association
DVL1/2 association
VANGL association
CK1 association
DVL1
DVL2
VANGL1
VANGL2
CK1ε
CK1δ
Enrichment in WT
TNKS1 association
TNKS2 association
CCDC88A association
CCDC88C association
Enrichment in phospho-switch mutants
ADGRA2 association
RYK association
RNF43 association
ZNRF3 association
TurboID (Euclidean Distance)
Charge

Figure S12. TurboID validation.
(**A**) Expression of TurboID hDVL3 variants induced by 1 μg/ml doxycycline, and TurboID biotinylation induced by 50 μM biotin, analyzed by western blotting. β-actin was used as the loading control. (**B**) Volcano plots showing specific interactions for WT and phospho-switch DVL3 mutants. Volcano plots show comparison between TurboID-DVL3 and TurboID only. Selected well-know DVL3 interactors acting in the Wnt pathway are highlighted. (**C**) The potency of selected proteins to interact with WT DVL3 and phospho-switch variants was calculated as the Euclidean distance from the origin of the volcano plots comparing the interactome of phospho-switch mutants with WT. The corresponding values were plotted as a function of the pIDR2 net charge (at pH=7.2) proximal to the DEP domain.

Figure S13

A

```
hDVL3(aa335-400) KCWDPSPRGCFTLPRSEPIRPIDPAAWVSHTAAMTGTFPAYGMSPSLSTITSTSSSITSSIPDTER
hDVL3 Δ S/T      KCWDPSPRGCFTLPRSEPIRPIDPAAWVSHTAAMTGTFPAYGMSP-----------------DTER
dDSH(aa338-388)  KCWDPNPKGYFTIPRTEPVRPIDPGAWVAHTQALT-SH----DSI-IADI------AE---PIKER
```

B

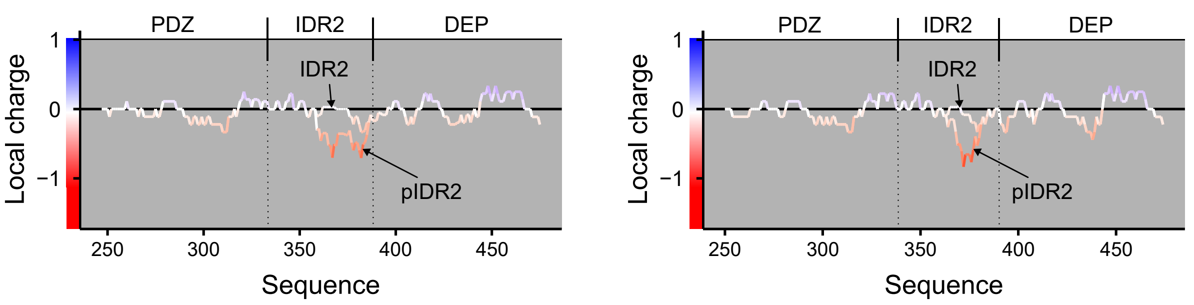

Figure S13. Δ S/T and dDSH similarities.
(**A**) Sequence alignment of IDR2 of human DVL3 (WT and Δ S/T mutant) and Drosophila melanogaster dDSH. Conserved S/T residues are highlighted in dark grey and other negatively charged residues in dDSH are shown in bold. (**B**) Local charge distributions of PDZ-IDR2-DEP and PDZ-pIDR2-DEP for hDVL3 Δ S/T and dDSH.

Table S1. Multiple sequence alignment of 854 DVL sequences from vertabrates.

Table S2. HDX data table.

Table S3. Proximity labelling data table.
